# No transfer of arousal from other’s eyes in Williams syndrome

**DOI:** 10.1038/s41598-023-45521-5

**Published:** 2023-10-26

**Authors:** Johan Lundin Kleberg, Astrid E. Z. Hallman, Martyna A. Galazka, Deborah M. Riby, Sven Bölte, Charlotte Willfors, Christine Fawcett, Ann Nordgren

**Affiliations:** 1 Department of Psychology, Stockholm University, Stockholm, Sweden; 2 Centre for Psychiatry Research, Department of Clinical Neuroscience, Karolinska Institute, Stockholm, Sweden; 3 Department of Applied Information Technology, Division of Cognition and Communication, University of Gothenburg, Gothenburg, Sweden; 4 Department of Psychology, Durham University, Durham, UK; 5 Center of Neurodevelopmental Disorders (KIND), Centre for Psychiatry Research, Department of Women’s and Children’s Health, Karolinska Institutet & Stockholm Health Care Services, Region Stockholm, Stockholm, Sweden; 6 Child and Adolescent Psychiatry, Stockholm Health Care Services, Region Stockholm, Stockholm, Sweden; 7 Curtin Autism Research Group, Curtin School of Allied Health, Curtin University, Perth, WA Australia; 8 Department of Molecular Medicine and Surgery, Karolinska Institute, Stockholm, Sweden; 9 Department of Psychology, Uppsala University, Uppsala, Sweden; 10 Sahlgrenska Academy, University of Gothenburg, Gothenburg, Sweden

**Keywords:** Human behaviour, Neuroscience, Psychology, Medical research

## Abstract

Typically developing humans automatically synchronize their arousal levels, resulting in *pupillary contagion,* or spontaneous adaptation of pupil size to that of others. This phenomenon emerges in infancy and is believed to facilitate social interaction. Williams syndrome (WS) is a genetic condition characterized by a hyper-social personality and social interaction challenges. Pupillary contagion was examined in individuals with WS (*n* = 44), age-parallel-matched typically developing children and adults (*n* = 65), and infants (*n* = 79). Bayesian statistics were used. As a group, people with WS did not show pupillary contagion (Bayes factors supporting the null: 25–50) whereas control groups did. This suggests a very early emerging atypical developmental trajectory. In WS, higher pupillary contagion was associated with lower autistic symptoms of social communication. Diminished synchronization of arousal may explain why individuals with WS have social challenges, whereas synchronization of arousal is not a necessary correlate of high social motivation.

## Introduction

Eye contact plays a crucial role in human social communication. In typically developing populations, eye contact facilitates emotion recognition and mental state understanding^1, 2^ and increases positive affect^3^. During mutual gaze, the pupil size of typically developing individuals adapt to that of the interacting partner so that larger pupils in others elicit greater dilation^4, 5^. This involuntary effect termed *pupillary contagion* is observed in typically developing populations as young as four months of age^6, 7^ and is relatively independent of age thereafter^8^. Pupil size is directly controlled by joint innervation from both branches of the autonomic nervous system (ANS)^9^ and pupillary contagion is therefore interpreted as being driven by spontaneous arousal synchronization^7^. In typically developing populations, pupillary contagion correlates with an increased activity in brain regions involved in mentalizing and social cognition including the superior temporal sulcus and medial prefrontal cortex^10^.

Arousal synchronization between humans may promote social closeness and interaction^11^. Consistently, pupillary contagion is found to facilitate mutual trust and empathy in typically developed adults and could therefore have an important role in social communication^4, 12^. If this is correct, altered pupillary contagion would be expected in neurodevelopmental conditions associated with differences in social interaction and eye contact. This theory remains largely unexamined.

The current study examined pupillary contagion in Williams syndrome (WS), a rare genetic disorder caused by a hemideletion of approximately 25 genes at chromosome 7q11.23. WS is associated with a striking behavioral phenotype, characterized by high social approach motivation, increased reward value of social stimuli^13^ and increased attention to other’s faces^14–17^. Because of its distinct phenotype and known genetic cause, WS has been seen as a model condition for studies of social brain development and function^18^. Many individuals with WS have an intellectual disability (ID) in the mild to moderate range^19^ and while variable, many require a high degree of societal support^20^. Despite a heightened social drive and interest in social interaction, people with WS often experience challenges in social communication and cognition, even relative to other populations with intellectual disability. These include social communication differences^21^, altered Theory of Mind^22, 23^ and challenges establishing social reciprocity^24^. People with WS often find it difficult to judge the trustworthiness of others, which can expose them to risks^25, 26^. Reduced facial emotion recognition skill has also been reported^27^, although the results are inconsistent^28^. The combination of high social drive and social skill challenges seemingly contrasts with influential theoretical accounts of neurodevelopmental conditions such as autism, where low social motivation might fuel social cognition and interaction alterations^29^.

Autism is a neurodevelopmental condition associated with atypical social interaction and eye contact^30, 31^. A recent study reported typical pupillary contagion associated with autism^32^ suggesting that reduced pupillary contagion is not a necessary correlate of neurodevelopmental conditions associated with *reduced* sociability. To better understand the role of pupillary contagion in human social interaction and motivation, it is crucial to determine whether it is atypical in rare developmental conditions associated with increased rather than reduced sociability.

Several studies have documented atypical attention to other’s eyes in individuals with WS, although the exact nature and consequences of these alterations remain unclear^33–36^. Ridley and colleagues^36^ analyzed parent reports of eye contact in WS and found descriptions of an unusual quality of eye contact in a large proportion of participants, including staring, and brief glances. Experimental studies reported altered attention to the eye region of static images of faces in WS which seem time-scale dependent. When visual attention is averaged over several seconds, people with WS were found to attend more to the eye region than controls^14, 34^ whereas a recent study including a relatively large sample with WS (*n* = 37) found a reduced tendency to orient to others’ eyes directly following stimulus onset^33^. A study using the standardized Autism Diagnostic Observation Schedule (ADOS)^37^, a first-choice clinical scale to assess autistic symptoms, reported unusual eye contact in 50% of young children with WS^38^. In the ADOS, eye contact is judged as being either typical or atypical based on the examiner’s impression of eye gaze being appropriate, flexible, socially integrated and modulated or not.

Several studies have documented differences in social perception and decision-making between people with WS and idiopathic autism, that is autism of unknown origin (e.g., no presence of a genetic syndrome)^15, 23, 27, 39, 40^. However, studies using the ADOS have documented elevated levels of autistic symptoms in WS^38, 41^, and may therefore explain inter-individual variability in social processing.

To the best of our knowledge, pupillary contagion has not been studied in WS. However, the unique combination of heightened social drive, atypical eye contact, and social cognitive challenges means that the condition is ideal for disentangling how pupillary contagion varies, based on genetic and neurodevelopmental conditions and in human development in general. Since pupillary contagion has previously been found to be relatively stable throughout development, we compared participants with WS not only to age-parallel-matched typically developing participants but also to typically developing infants aged 4 to 6 months, i.e., the earliest age when pupillary contagion is consistently found. These groups were included to address some of the challenges associated with the selection of comparison groups in studies of rare genetic conditions associated with intellectual disability. Age-matched typically developing controls will differ from study participants on multiple parameters, including cognitive. Controls matched for cognitive ability will typically be much younger and therefore also differ from the study group on multiple parameters. Finally, a comparison group with intellectual disability of unknown genetic origin may be included. This avoids some of the problems with typically developing control groups but is also likely to result in a comparison group with high but unknown genetic heterogeneity, since most cases of intellectual disability in the moderate to mild range are known to be genetic^42^.

## Methods

### Stimuli

**Figure 1:**
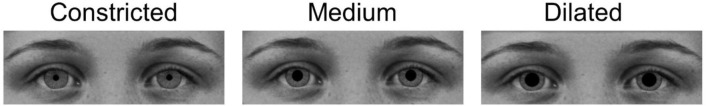
Example of stimulus images.

Images of the eye region of human faces with a neutral emotional expression (see Fig. 1) were presented. The original images were taken from the Karolinska Directed Emotional Faces database^43^ and digitally edited to create three versions of each image with different pupil sizes, henceforth referred to as *constricted, medium,* and *dilated.* Constricted pupils were 40% smaller than medium pupils, which were in turn 40% smaller than dilated pupils. The three stimulus categories had identical mean image brightness^6^ and covered 21.74° of the visual field horizontally and 8.03° vertically. The full stimulus set consisted of images of six models (3 males, 3 females) (Image IDs: BF19NES, BF13NES, BF01NES, AM14NES, AM10NES, and AM08NES), editing described in Ref.^6^. For examples, see Fig. 1.

### Procedure

Participants completed one of two versions of the task. The long version included images of all six models which were presented twice with each pupil size, resulting in 36 trials, following the original study where the stimuli were used^6^. The short version of the task included four of the six models (3 male, 1 female) presented once with each pupil size, resulting in 12 trials. Data collection started using the shorter version of the task to minimize the attentional demands of participants. Qualitative observations during testing indicated that the task was not too demanding for most participants. To facilitate comparisons with the first study using the stimuli^6^, and increase the number of data points, we therefore switched to the longer version of the task. Stimuli were shown for 3 s in counterbalanced order, preceded by a fixation cross shown for one second. Data collection took place at a lab facility at the Karolinska Institute, at the Uppsala University Child and Baby Lab (infants), or at the Ågrenska Foundation in Gothenburg, Sweden. The study is part of a larger project which examines the behavioral, genetic, neural, and medical aspects of rare genetic disorders^13, 33, 44^.

### Participants

The study included individuals with WS and two comparison groups: typically developed children and adolescents, and typically developing infants. Since WS is a rare condition, it was not deemed feasible to determine a sample size a priori. Instead, recruitment was conducted continuously and stopped when information about the study had been spread through all available channels (see *Williams syndrome*) and when the sample size was comparable to previous studies finding a pupil contagion effect^6, 7, 32, 45^. Sample size in the comparison groups was determined based on previous studies of pupil contagion in typically developing individuals^5,7, 45, 46^.

Demographic information, baseline pupil size, and number of included trials by group and condition are shown in Table 1. TD-infants were (by definition) younger than the other groups. No other differences were found.

**Table 1:** Demographic characteristics and number of valid trials.

	WS (n = 44)	TD (n = 65)	TD-infant (n = 79)	Group differences‡
Age (M, SD)	25.43 (13.48)	31.04 (12.92)	0.48 (0.1)	WS, TD > TD-infant (BF_10_ > 3, p < 0.001)
%Female	48%	58%	53%	
ADOS-2 total (CSS)	1.71 (0.78)	–	–	
ADOS-2 Social affect (CSS)	2.33 (1.15)	–	–	
ADOS-2 RRB (CSS)	2.95 (2.25)	–	–	
Valid trials (M, SD)				
Constricted	5.91 (4.08)	5.63 (3.51)	6.38 (3.73)	
Medium	5.93 (3.94)	5.6 (3.59)	6.24 (3.58)	
Dilated	5.86 (3.92)	5.6 (3.55)	6.57 (3.7)	
Baseline pupil size in mm (M, SD)				
Constricted	3.71 (0.7)	3.38 (0.58)	3.7 (1.06)	TD-infant > TD (BF_10_ = 1.47; p = 0.026)
Medium	3.77 (0.7)	3.43 (0.62)	3.64 (1.17)	
Dilated	3.71 (0.67)	3.36 (0.59)	3.66 (1.07)	TD-infant > TD (BF_10_ = 1.05, p = 0.041)

*WS* Williams syndrome, *TD* Typically developed adults and children, *TD-inf* Typically developing infants, *BF*_*10*_ Bayes factor favoring a group difference.

^‡^Group differences were analyzed with t-tests for continuous variables and with Chi-square tests for gender proportions.

### Williams syndrome

Individuals with WS (*n* = 44, age range 9–57 years) were recruited through contacts with Habilitation and Health Care services, the Swedish Williams Syndrome Association, and the Ågrenska Foundation. Five additional participants were tested but excluded because of invalid data. Protocols from genetic assessments were available for 22 participants. These had typical deletions at the 7q11.23 region. In individuals not genetically tested in the current study, a diagnosis of WS was confirmed from medical records and caregiver report (*n* = 4) or parental report only when medical records could not be obtained (*n* = 18). Demographic characteristics are shown in Table 1. Fifteen of the 44 participants completed the longer version of the task.

### Psychiatric assessment

Twenty-two individuals, and their caregivers, completed a larger assessment including the Wechsler Adult Intelligence Scale, 4th Edition (WAIS-IV^47^) or the Wechsler Intelligence Scale for Children, 5th Edition (WISC-V^48^) depending on their age, and a diagnostic interview for psychiatric disorders conducted by a clinical psychologist or psychiatrist using the Mini International Neuropsychiatric Interview (MINI)^49^. Co-occurring psychiatric conditions were specific phobia, *n* = 8, Attention deficit/hyperactivity disorder (ADHD), *n* = 3, panic disorder, *n* = 4, post-traumatic stress disorder (PTSD), *n* = 1, tics disorder, *n* = 1, depression, *n* = 1, autism, *n* = 1. These participants completed the ADOS-2 with module selected depending on the participant's age and verbal ability (Module 2: n = 1, Module 3: n = 3; Module 4: n = 4). ADOS-2 gives separate scores for the social communication (termed social affect) and restricted and repetitive behavior (RRB) domains of autistic symptoms as well as a total score. Following^50^, raw scores were converted to calibrated severity scores (CSS) to facilitate comparisons across ADOS-2 modules.

*Typically developing children and adults* (TD, age range 9 to 63 years) were recruited from a database of individuals expressing interest in participating in research and through advertising in social media. Exclusion criteria were any ongoing psychiatric or neurological condition. Initially, 66 individuals expressed an interest and participated. Of these, the absence of an ongoing psychiatric disorder was confirmed by an experienced clinical psychologist using the MINI in 51 cases, and by self-report in 15^49^. Of the initial sample, one was excluded because of a lack of valid data, resulting in a final sample of 65 individuals of which 50 completed the short, and 15 the long version of the task.

*Typically developing infants* (TD-infant, age range 4 to 6.5 months) were recruited through a database of families expressing interest in participating in developmental research. The final sample consisted of 79 infants of which 31 completed the short, and 48 the long version of the task. In addition, eight participants were seen but excluded because one parent had a diagnosis of a neurodevelopmental condition (*n* = 4), gestational age less than week 35 (n = 1), birth complications (n = 1), suspected genetic syndromes in multiple second-degree relatives (n = 1), or low data quality (*n* = 16). Infants completing the shorter version of the task were recruited specifically for the current study, whereas the group completing the longer version were tested in the context of a previous study using the same stimuli^6^.

### Ethical approval and consent to participate

The study was conducted in accordance with the declaration of Helsinki and approved by Regional Ethics Committee of Stockholm, Sweden (dnr 2018/1218-31 with subsequent amendments). Participants aged 15 or above gave verbal and written consent to participate. Legal guardians of participating children gave written consent.

### Pupil dilation recording and analysis

Data were recorded using Tobii corneal reflection eye trackers (Tobii Inc., Danderyd, Sweden). Eighty-two participants (TD, n = 50, WS, n = 29) were tested with a Tobii X-120 which samples the pupil at 40 HZ and gaze at 120 HZ, 30 participants with a Tobii Spectrum (TD, n = 15, WS, n = 15) at 1200 HZ. The infant group was tested with a Tobii T120 at 60 HZ (n = 48) or a TX300 at 120 HZ (n = 31).

Data were first downsampled to 40 HZ. Subsequently, gaps in the data shorter than 8 samples were interpolated. Samples directly preceding and succeeding an absolute sample-to-sample change in pupil size > 3* the median absolute distance (MAD) were removed, since sudden changes of this magnitude are physiologically unlikely. After that, data were filtered with a moving average filter covering 8 samples. Initial visualization of the data (see Fig. 3) showed a pupillary light reflex caused by change in luminance at stimulus onset approximately within the 0–1.5 s time period following stimulus onset. Since the pupillary light reflex is not sensitive to attention and has other neural correlates than task-evoked pupil dilation^9^, this interval was removed from analysis in line with previous studies^5, 8^. The pupil dilation response was defined as the peak pupil dilation during the 1.5–3 s interval, baseline corrected by subtracting mean pupil size during the last 0.4 s of the preceding fixation cross to correct for differences in tonic pupil size^6^.

Trials with < 30% valid samples during the baseline or trial interval were rejected (WS: 11.80%, TD: 3.68%, TD-infant: 28.10%). To maximize the number of included data points, all valid trials were included, i.e., participants were not excluded if they only contributed a small number of trials. However, results did not change when participants completing less than two valid trials per condition were excluded. Saccades were identified with an I-VT filter algorithm with a velocity threshold set to 25°/second. Fixations were defined as periods between saccades. Subsequent fixations at a distance shorter than 0.5° and occurring within 75 ms were merged. Eye movement data were analyzed at the original sampling rate.

### Statistical analysis

Data were analyzed using Bayesian linear mixed effects models (LMM) with random effects for individual. LMMs model simultaneously model data at the levels of trial and participant, which is preferable to traditional analysis of variance (ANOVA) in designs with multiple trials per participants and potential imbalance between conditions in the number of included trials due to data loss^51^. Perhaps most importantly for the current study, LMMs do not assume an equal number of trials per participants and can account for potential variation between stimuli within a condition by specifying random factors. The statistical models included pupil dilation as dependent variable and trial number, percent gaze recorded during the trial, sex, and age as covariates. Random factors were participant id (i.e., trials from the same individual were treated as repeated measures) and stimulus model. Eye tracker model was included as an additional covariate in the WS and TD groups, but not in infants since all infants tested at 120 HZ were older than four months, leading to multicollinearity between age and eye tracker.

A set of exploratory analyses examined the relationships between individual estimates of the pupillary contagion effect and symptoms of autism and IQ. Participants with four or more valid trials in the medium and dilated conditions and available clinical data were included in these analyses (n = 16, note that 22/44 participants completed the longer clinical assessment described under *Psychiatric Assessment*). Visualizations of the ADOS-2 total and RRB scores indicated a bimodal distribution, and they were therefore transformed to a binary variable with two levels: no- to minimal symptoms (0–1 points) or symptoms present (2 or above). Continuous variables were screened for outliers defined as values outside the median + /− 3*MAD range. This threshold for outlier detection can be considered as “very conservative”^52^. One outlier value in ADOS-2 Social Affect was detected and replaced by the highest acceptable value.

We reasoned that evidence for a *lack* of pupillary contagion in WS would be equally informative as evidence for it, as a lack of pupillary contagion would indicate a potentially important deviation from normative development. Because of this, we chose to use Bayesian statistics for the current analysis. Bayesian statistics directly compare the likelihood of the hypothesis (e.g., that a pupillary contagion effect exists) to that of a null hypothesis (e.g., that no contagion effect exists). Therefore, three potential conclusions are possible: (1) that the *null hypothesis is best supported,* (2) that the *hypothesis* is best supported, and (3) that the data is inconclusive. This contrasts with traditional null hypothesis significance testing (NHST) based on *p-*values. In NHST, a result can only indicate that the null hypothesis should, or should not be rejected, but not the degree to which it is supported, even if *p-*values are high. In studies of rare genetic disorders, it may be particularly important be able to differentiate between *support for the null hypothesis* and *insufficient support for the hypothesis* (is there evidence for absence of an effect, or absence of evidence?). Specifically for our study, we tested whether there is evidence to support the existence of pupillary contagion in WS (*H1*), evidence to support its absence (*H0*), or whether the results are inconclusive.

Statistical decisions were based on Bayes factors (BF_10_) which quantifies the relative support for the hypothesis over the null. By convention, a BF_10_ > 3 indicates support for the hypothesis, BF_10_ > 20 strong support, and BF_10_ > 150 very strong support. A BF_10_ > 0.5 and < 3 indicates inconclusive results^53^. A BF_10_ can be converted to reflect the degree of support for the null (BF_01_) through the formula 1/BF_10_ which can be interpreted at the same scale. Since the pupillary contagion effect implicates *larger* pupil dilation to relatively larger pupils, Bayes factors are expressed for directed hypotheses. To facilitate comparison with traditional null hypothesis significance testing, we report *p-*values based on the probability of direction (pd) measure. *Pd* ranges between 0.5 and 1 and indicates the degree to which the posterior distribution goes in the observed direction. The value 1-pd is equivalent to a one-sided *p*-value^54^.

Bayesian parameter estimation results in a distribution of plausible parameter values in which a highest density interval (HDI) can be defined, which covers the most plausible values. Following^55^, we compared the 95% HDI (the credibility interval, CI) of the estimated parameters to a region of practical equivalence (ROPE) covering 0 + /0.1 SD of the dependent variable corresponds to a small effect size^56^. Overlap between the ROPE and the HDI indicate that the parameter value is practically equivalent to zero. The package *rstanarm*^57^ in *R*^58^ was used to fit all models. The BayestestR library was used to calculate Bayes factors, 95% HDIs and ROPE intervals. Weakly informative Cauchy priors (μ = 0, scale = 0.707) were used in all analyses. Visualizations indicated that the residuals from all models were approximately normally distributed. R-hat values > 0.999 for all parameters, indicating good model convergence.

## Results

### Covariates

**Figure 2:**
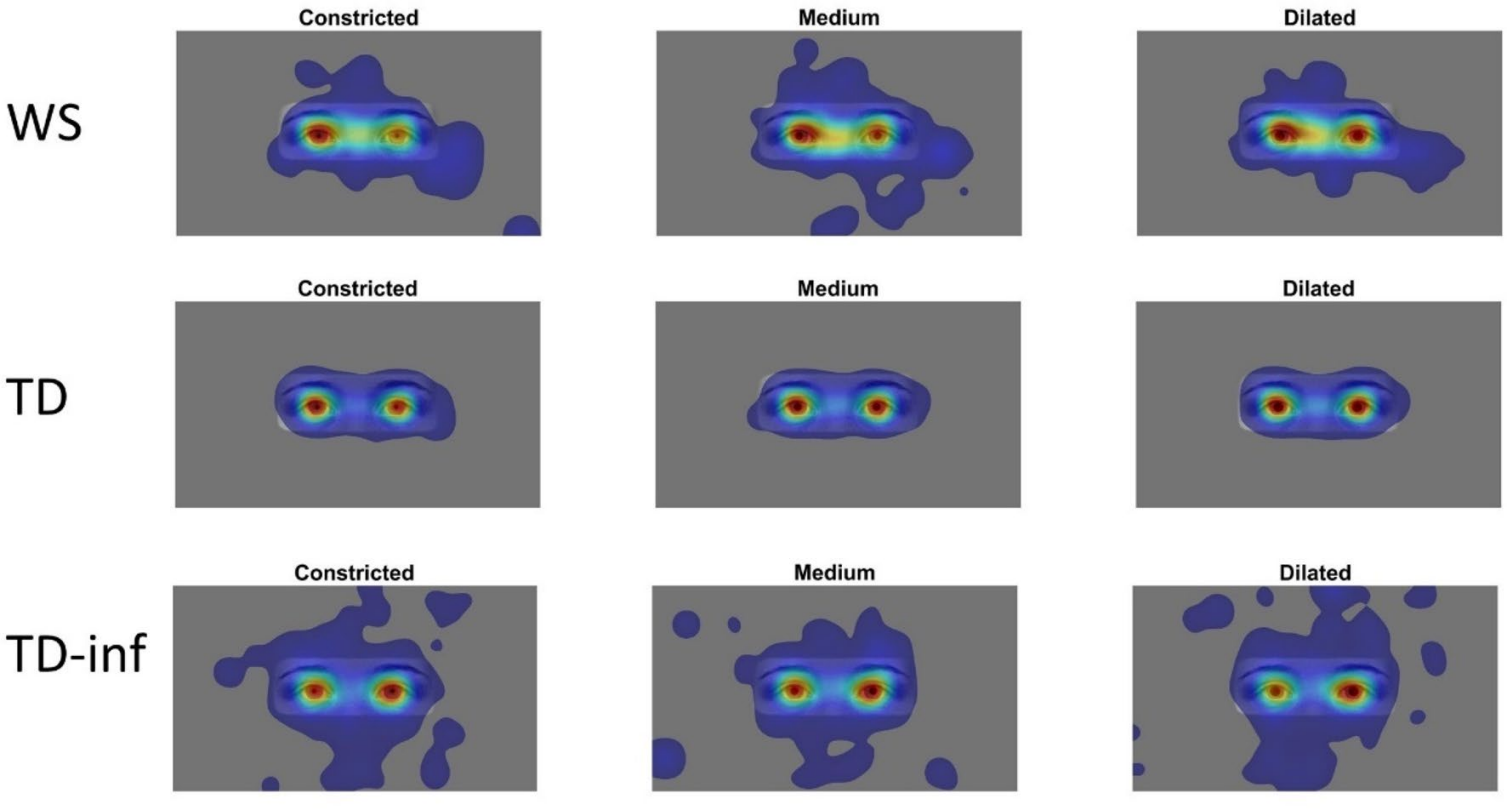
Heatmaps of gaze allocation to constricted, medium sized, and dilated pupils by group. Gaze data were smoothed with a Gaussian filter with sigma corresponding to 1 degree of the visual field. The scale goes from blue to red, with more gaze allocations at more red areas.

Looking time at the stimuli (%gaze) was not associated with pupil dilation in any of the groups (BF01 > 3), see Fig. 2). Higher age was associated with lower pupil dilation in typically developing children and adults (BF_10_ = 10.24) with a small effect size, indicated by almost complete overlap with the ROPE. No relation between pupil dilation and age was seen in the other groups (BF_10_ < 1). Other covariates were not associated with pupil dilation (all BF_10_ < 2.7, see Table 2).

**Table 2:** Results of Bayesian linear effect models in typically developing groups.

Parameter	b	CI (lower)	CI_(upper)	pd	p	% in ROPE	BF_10_	BF_01_
Typycally developing children and adults								
Dilated > medium	** − 0.051**	** − 0.081**	** − 0.020**	**1.000**	< 0.001	**1.60**	**17.69**	**0.06**^‡^
Dilated > constricted	− 0.037	− 0.067	− 0.006	0.990	0.010	17.96	1.20	0.83^‡^
Medium < constricted	0.014	− 0.016	0.045	0.817	0.183	18.30	0.05	19.21
Trial	− 0.001	− 0.003	0.001	0.911	0.089	100.00	0.1	9.88
Sex	− 0.027	− 0.087	0.033	0.809	0.191	43.16	0.11	9.18
Age	− 0.004	− 0.006	− 0.001	0.999	0.001	100.00	10.24	0.1
Eyetracker	0.079	0.001	0.157	0.976	0.024	5.91	0.75	1.34
%gaze	− 0.011	− 0.056	0.034	0.676	0.324	67.72	0.04	25.03
Typically developing infants								
Dilated > medium	** − 0.052**	** − 0.086**	** − 0.017**	**0.998**	0.002	**8.85**	**5.23**	**0.19**^‡^
Dilated > constricted	− 0.033	− 0.067	0.002	0.968	0.032	44.78	0.36	2.78^‡^
Medium < constricted	0.019	− 0.054	0.0151	0.864	0.136	74.66	0.06	16.67
Trial	0.000	− 0.002	0.001	0.620	0.380	100.00	0.03	32.53
Sex	− 0.017	− 0.086	0.054	0.686	0.314	58.92	0.08	13.3
Age	0.478	0.138	0.815	0.996	0.004	0.00	2.7	0.37

*pd* probability of direction, *ROPE* region of practical equivalence, *BF*_*10*_ Bayes factor supporting the hypothesis, *BF*_*10*_ Bayes factor supporting the null hypothesis.

P-values are computed according to the formula 1-pd and correspond to one-sided p-values. Bayes factors > 3 and p-values < 0.05 are shown in bold.

**p < 0.01.

*p < 0.05.

^‡^Bayes factor for the directed hypothesis (Dilated > Medium and Dilated > Constricted).

### Main analysis: typically developing groups

**Figure 3:**
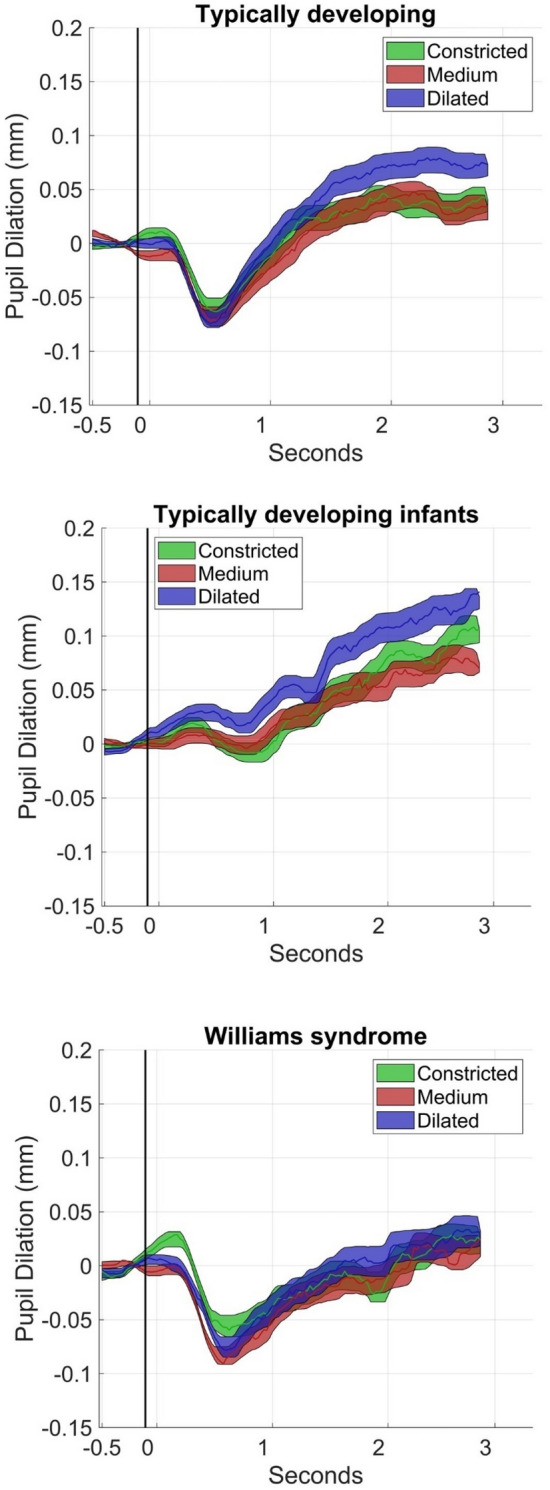
Mean pupil dilation while viewing constricted, medium, and dilated pupils as a function of time split by group. Shaded areas cover + / − 1 standard error of the mean.

As expected, higher pupil dilation to eyes with dilated as compared to medium pupils was found in typically developing children and adults (BF_10_ = 17.69) and infants (BF_10_ = 5.23), see Fig. 3. As can be seen in Table 2, the posterior distributions had only a small overlap with the 95% ROPE, providing further evidence for the hypothesis. Evidence for a difference between dilated and constricted pupils was inconsistent in both typically developing groups (children and adults: BF_10_ = 1.20, infants: BF_10_ = 0.36). However, it can be noted that the estimated effect was in the expected direction with a one-sided p-value of *p* = 0.01 for children and adults and *p* = 0.032 for infants.

### Main analysis: Williams syndrome

The data strongly supported the null hypothesis that pupil dilation responses did not differ between conditions (all BF_10_ ≤ 0.04, all BF_01_ ≥ 25.00, see Fig. 3). As seen in Table 3, the posterior distributions of the effects had large overlaps with the ROPE, suggesting that the effect sizes can be considered practically equivalent to zero.

**Table 3:** Results of Bayesian linear effect models in Williams syndrome.

Parameter	b	CI (lower)	CI (upper)	pd	% in ROPE	p	BF_10_	BF_01_
Williams syndrome								
Dilated > medium	0.002	− 0.034	0.038	0.548	80.35	0.452	0.04	25.00^‡^
Dilated > constricted	0.018	− 0.018	0.054	0.840	58.35	0.160	0.02	50.00^‡^
Medium < constricted	0.016	− 0.019	0.052	0.812	18.80	0.188	0.07	14.83
Trial	− 0.001	− 0.003	0.001	0.897	100.00	0.103	0.1	9.58
Sex	0.036	− 0.017	0.086	0.916	27.16	0.084	0.19	5.25
Age	− 0.002	− 0.004	0.000	0.974	100.00	0.026	0.46	2.17
Eyetracker	0.011	− 0.041	0.062	0.661	58.84	0.339	0.07	14.44
%gaze	− 0.015	− 0.066	0.035	0.724	55.16	0.276	0.06	16.71

*pd* probability of direction, *ROPE* region of practical equivalence, *BF*_*10*_ Bayes factor supporting the hypothesis, *BF*_*10*_ Bayes factor supporting the null hypothesis.

P-values are computed according to the formula 1-pd and correspond to one-sided p-values. Bayes factors > 3 and p-values < 0.05 are shown in bold.

**p < 0.01.

*p < 0.05.

^‡^Bayes factor for the directed hypothesis (Dilated > Medium and Dilated > Constricted).

### Relationship between pupil contagion, autistic symptoms, and IQ in Williams syndrome

**Figure 4:**
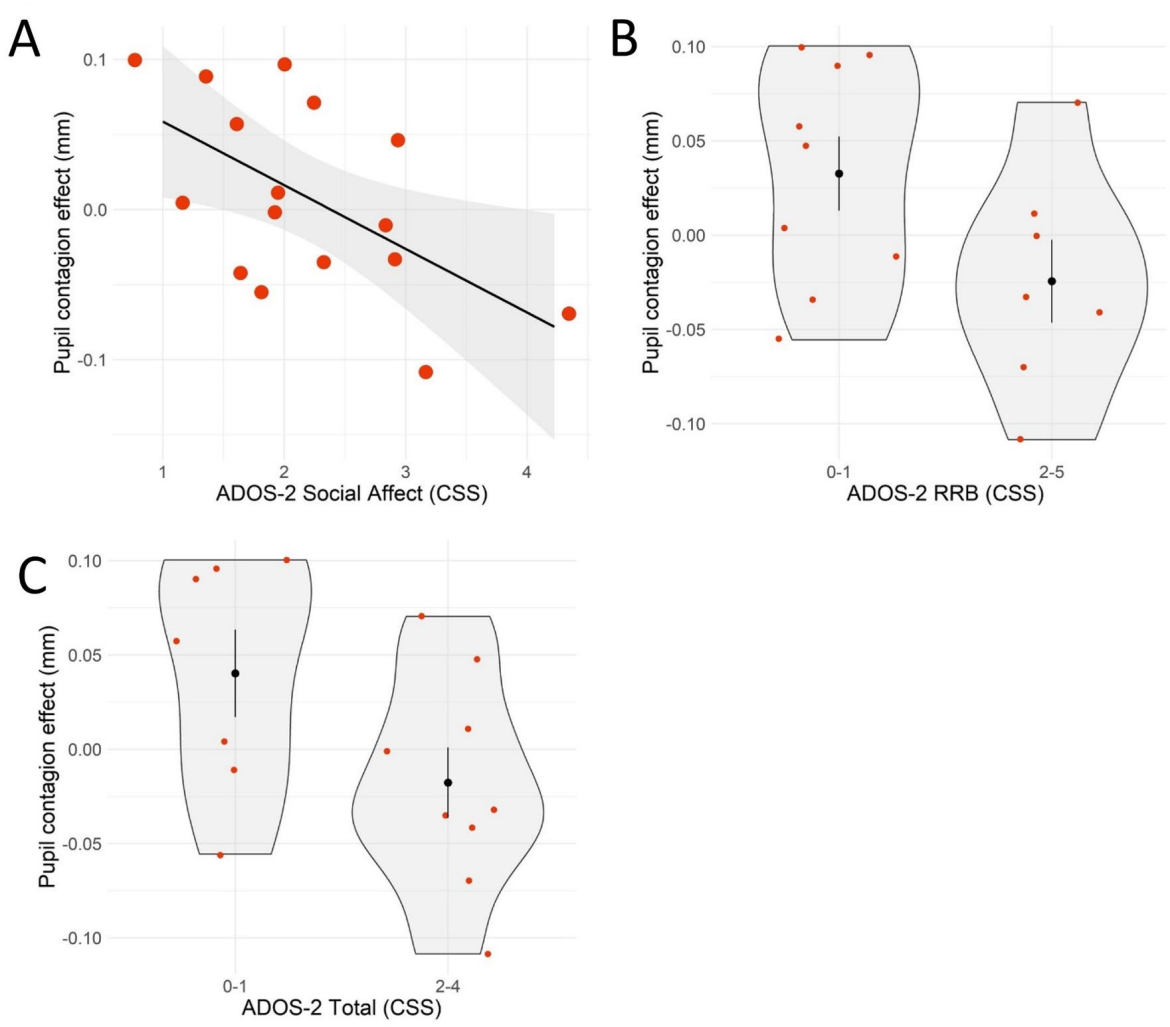
Relationship between pupillary contagion and ADOS-2 measures of autistic symptoms of social affect (**A**), repetitive behaviors and restricted interests (**B**), and ADOS-2 Total scores (**C**) in individuals with Williams syndrome. (**B**,**C**) show dichotomous variables due to a bimodal distribution.

A negative relationship was found between ADOS-2 Social Affect and pupillary contagion (b =  − 0.037, CI =  − 0.071, 0.004, BF_10_ = 3.278, p = 0.028, see Fig. 4 and Table 4), indicating that one point increase in social communication symptoms corresponded to a 0.037 mm lower pupillary contagion effect, approximately 75% of the pupillary contagion effect seen in TD-adults and children. In contrast, inconclusive evidence for a relationship between pupillary contagion and ADOS-2 total and RRB scores and IQ were found (see Fig. 4 and Table 4).

**Table 4:** Relationship between pupillary contagion and clinical and demographic factors in Williams syndrome.

Parameter	b	CI (higher)	CI (lower)	pd	%in ROPE	p (two-sided)	BF_10_	BF_01_
ADOS-2								
Total	− 0.049	− 0.108	0.009	0.953	4.1	0.094	1.225	0.816
Social affect	− 0.037	− 0.071	− 0.004	0.986	0.9	0.028*	**3.054**	0.327
RRB	− 0.049	− 0.109	0.01	0.952	4.5	0.096	1.106	0.904
IQ	0.002	− 0.001	0.005	0.878	100	0.244	0.575	1.739

*pd* probability of direction, *ROPE* region of practical equivalence, *BF*_*10*_ Bayes factor supporting the hypothesis, *BF*_*10*_ Bayes factor supporting the null hypothesis.

P-values are computed according to the formula (1-pd)*2 and correspond to two-sided p-values. Bayes factors > 3 and p-values < 0.05 are shown in bold.

**p < 0.01. Analyses based on n = 16.

## Discussion

Pupillary contagion is a developmentally early emerging phenomenon believed to facilitate social affiliation and group interaction. The current study examined pupillary contagion in WS, a condition associated with a combination of a hypersocial behavioral phenotype and social interaction challenges. The data gave strong support for the null hypothesis that, at the group level, pupillary contagion is *not* seen in WS. Although the null hypothesis was supported, there was considerable variability between individuals. A follow-up analysis showed that this variability was partly explained by autistic symptoms of social communication, so that individuals with lower symptom levels showed a more normative pupillary contagion effect.

Taken together, these results suggest that high social drive, a cardinal feature of WS, does not necessarily result in spontaneous synchronization of arousal through the eyes. Instead, lack of spontaneous synchronization of arousal may be part of the underlying mechanism of social communication challenges faced by individuals with WS. For example, people with WS often have challenges judging the trustworthiness of others^4, 59^, an ability previously linked to pupil contagion^4^.

The present results may also partly explain why a high level of interest in others’ eyes as commonly seen in WS does not lead to social interaction skills. As noted in the introduction, studies examining attention to others’ eyes over periods ranging from seconds to minutes demonstrated a high level of attention to this region^14, 17, 34^. In a striking contrast, studies examining the time scale^33^ and communicative use^36, 38^ of eye contact in people with WS demonstrated clear differences from the typical developmental pattern. This underlines the importance of treating social attention in neurodevelopmental conditions as a multifaceted concept involving multiple cognitive and attentional mechanisms^60^.

Replicating previous studies, pupillary contagion was found in neurotypical comparison groups from four-month-old infants to adults completing the same task. This suggests that the reduced pupillary contagion effect found in WS represents an altered developmental trajectory at a very early stage. It should be noted that Bayesian statistics directly quantifies the support for the null, and that the null result in WS does not merely indicate inconclusive data, as would be the case with a high p-value. In the present study, a negative relationship between pupillary contagion and social communication symptoms was seen with an observed effect size of 0.37 mm (CI − 0.037, − 0.071) corresponds to approximately 75% of the average pupillary contagion effect observed in typically developing children and adults, suggesting that a practically meaningful part of the individual variability was explained. Only a subset of 16 individuals were available for this analysis. Although this small subsample merits caution, it is on par with the majority of previous eye tracking studies of WS. It should also be noted that Bayesian statistics are more robust to false positives than traditional null hypothesis significance testing^53^.

Although the neural mechanisms underlying diminished pupil contagion in WS remain to be understood, it should be noted that functional and structural atypicalities have been noted in medial prefrontal brain regions in WS^18, 61^ which have also been suggested to underlie pupil contagion in typically developing adults^10^. Atypical tonic and phasic activity in the ANS have also been suggested in previous studies.

Although the current study included one of the largest groups with WS in experimental research, it should be noted that the age range was wide. We were also not able to examine the longitudinal predictive relationships between pupillary contagion and later outcome. While the present study demonstrates altered pupillary contagion in WS compared to typically developing population across the development, additional studies involving genetically homogeneous populations with intellectual disability are needed to examine whether this is a syndrome-specific alteration.

The current study suggests that reduced synchronization of arousal may be one of the reasons why people with WS often have challenges with social interaction, despite their hyper-social personality and strong approach motivation. It is also in line with previous suggestions that, despite its hypersocial phenotype, WS is linked to altered processing of other’s eyes^33, 35^. These results contribute to our understanding of human sociability.

## Data Availability

The datasets generated and/or analyzed during the current study are not publicly available since participants did not give their consent to have data uploaded to a public repository. Anonymized data are available from the corresponding author on reasonable request.
